# *Megasphaera vaginalis* sp. nov. and *Anaerococcus vaginimassiliensis* sp. nov., new bacteria isolated from vagina of French woman with bacterial vaginosis

**DOI:** 10.1016/j.nmni.2020.100706

**Published:** 2020-06-03

**Authors:** A. Bordigoni, C.I. Lo, E.K. Yimagou, K. Diop, B. Nicaise, D. Raoult, C. Desnues, F. Fenollar

**Affiliations:** 1) Aix Marseille Université, IRD, AP-HM, MEФI, Marseille, France; 2) IHU-Méditerranée Infection, Marseille, France; 3) Aix Marseille Université, IRD, AP-HM, SSA, VITROME, Marseille, France

**Keywords:** *Anaerococcus vaginimassiliensis* sp. nov., Bacteria, Culturomics, *Megasphaera vaginalis* sp. nov., Taxonogenomics, Vagina

## Abstract

Using the culturomics method, two strains were isolated, identified and characterized following the taxonogenomics concept. *Megasphaera vaginalis* sp. nov. strain Marseille-P4512 (= CSURP4512) and *Anaerococcus vaginimassiliensis* sp. nov. strain Marseille-P4857 (= CSURP4857) were isolated from the vagina of a French woman. The phylogenic tree, phenotypic criteria and genomic analysis described here clearly show that these two bacteria are different from previously known bacterial species with standing in nomenclature and new members of *Firmicutes* phylum.

## Introduction

Healthy vaginal microbiota is a complex dynamic ecosystem, mainly dominated by *Lactobacillus* spp. and classified into five community state types (CST) depending on the following majority species: CST I (*Lactobacillus crispatus*), CST II (*Lactobacillus gasseri*), CST III (*Lactobacillus iners*) and CST V (*Lactobacillus jensenii*) [1,2]. These beneficial bacteria are the first line of defence against vaginal pathogens through competition and production of inhibitory compounds [3,4]. Bacterial vaginosis is a common infection due to an imbalance of the vaginal flora with an increase in CST IV, which is represented by anaerobic pathogenic bacteria, such as *Atopobium* sp., *Gardnerella* sp. and *Sneathia* sp.

The development of culturomics, combined with taxonogenomic analysis, has enabled the description of many previously unknown bacterial species [5,6]. Thanks to this strategy, our laboratory has characterized several new bacteria isolated from the vagina [[7], [8], [9]].

*Megasphaera* and *Anaerococcus* genera, respectively, belong to the *Veillonellaceae* and *Peptoniphilaceae* families within the *Firmicutes* phylum. At the time of writing and among validly published names, there are nine species described in *Megasphaera* and 13 species described in *Anaerococcus* [10]. Members of the *Megasphaera* genus, described in 1971 by Rogosa [11], can be found in human faecal flora [12,13], the mammalian digestive tract [14] and brewery samples [15]. Some *Anaerococcus* spp. were isolated from human clinical samples [16,17]. Among the 13 *Anaerococcus* species validly published, six were isolated from vaginal discharge or ovarian abscess samples: *Anaerococcus hydrogenalis*, *Anaerococcus lactolyticus*, *Anaerococcus vaginalis*, *Anaerococcus prevotii*, *Anaerococcus tetradius* and *Anaerococcus provencensis* [16,18].

We report here the description of two new designated species, *Megasphaera vaginalis* sp. nov. strain Marseille-P4857 and *Anaerococcus vaginimassiliensis* sp. nov. strain Marseille-P4512, belonging to the *Firmicutes* phylum.

## Material and methods

### Strain isolation and identification

As part of a culturomic study investigating the human microbiome, we isolated two bacterial strains from vaginal swabs of a French woman with bacterial vaginosis. These were strains Marseille-P4857 and Marseille-P4512. The patient provided informed consent, and the study was authorized by the ethics committee of the Institut Federatif de Recherche IFR48 under the number 09-022. The vaginal swabs were directly seeded in Petri dishes containing 5% sheep blood agar (BioMérieux, Marcy l'Étoile, France) and incubated under anaerobic condition (Thermo Scientific, Dardilly, France) at 37°C after 3 days.

Identification was performed with matrix-assisted laser desorption/ionization time-of-flight mass spectrometry (MALDI-TOF MS) (Bruker Daltonics, Bremen, Germany) as previously reported [19]. The spectra generated were analysed by Biotyper 3.0 software, which is regularly incremented with the local URMS database (https://www.mediterranee-infection.com/urms-data-base). Misidentification with MALDI-TOF MS led to amplification of the 16S rRNA gene using the primer pair fD1 and rP2 (Eurogentec, Angers, France) and then sequencing using the Big Dye® Terminator v1.1 Cycle Sequencing Kit and 3500xLGenetic Analyzer capillary sequencer (Thermofisher, Saint-Aubin, France), as previously reported [20]. All 16S rRNA nucleotide sequences were assembled and edited using CodonCode Aligner software (http://www.codoncode.com). Once a consensus sequence is obtained, it is submitted to the NCBI nucleotide database (https://www.ncbi.nlm.nih.gov/nucleotide/) and a comparative analysis of nucleotides by BLASTn (https://blast.ncbi.nlm.nih.gov/Blast.cgi?PROGRAM=blastn&PAGE_TYPE=BlastSearch&LINK_LOC=blasthome) is performed. Hence, the sequences phylogenetically closest to the only typical species are recovered to build the phylogenetic trees.

### Phenotypic characterization

Different growth conditions were tested for strains in aerobic, microaerophilic and anaerobic atmospheres (Thermo Scientific, Dardilly, France). The optimal temperature of growth was assessed (28°C, 37°C, 45°C and 55°C) on 5% sheep blood-enriched Columbia agar medium (BioMérieux). According to the manufacturer's recommendations, API ZYM and API 50 CH strips (bioMérieux) were employed to assess the biochemical characteristics of each strain. Phenotypic tests, such as Gram-staining, catalase and oxidase were performed. Also, the spore-forming was searched for each strain as previously reported [21]. The morphological structure of these two isolates was highlighted with a scanning electron microscope (Hitachi High-Technologies, Tokyo, Japan) following the protocol described by Belkacemi et al. [22].

### Genome characteristics

Genomic DNA extraction was performed with the EZ1 biorobot using the EZ1 DNA tissue kit (Qiagen, Hilden, Germany), and sequencing was performed on the MiSeq instrument (Illumina Inc., San Diego, CA, USA) using the Nextera Mate Pair and Nextera XT Paired End (Illumina) sample preparation kit, as previously described [20]. The genomic assembly was carried out using the three following softwares: Velvet [23], Spades [24] and Soap Denovo [25]. MiSeq and Trimmomatic [26] softwares were used for trimmed or untrimmed sequences. To reduce assembly gaps, GapCloser software [27] was used. Best assembly was determined using different criteria, such as the number of scaffolds, N50 or number of N. Scaffolds were deleted when their nucleotide number was <800 bp and their depth value < 25% of the mean depths. Genome annotation of these two species was performed as described elsewhere [28]. In addition, the Genome-to-Genome Distance Calculator web server available online (http://ggdc.dsmz.de) made it possible to assess the similarity between the genomes being compared and to replace the DNA–DNA hybridization (DDH) with a digital DDH (dDDH) [29]. Average nucleotide identity analysis was also evaluated using the OAT software [30].

## Results

### Strain identification and phylogenetic analysis

**Fig. 1:**
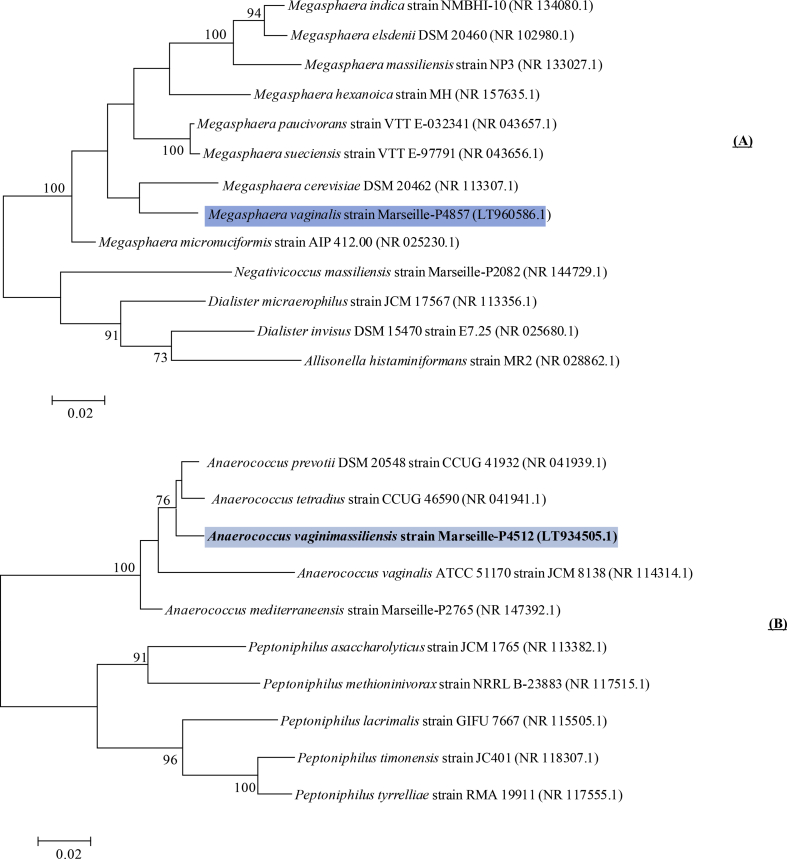
Phylogenetic trees displaying the position of *Megasphaera vaginalis* strain Marseille-P4857^T^ (a) and *Anaerococcus vaginimassiliensis* strain Marseille-P4512^T^ (b) relative to their closest phylogenetically related species. The respective GenBank accession numbers for 16S rRNA genes are indicated in parenthesis. Sequence alignment and phylogenetic inferences were obtained using the maximum likelihood method within MEGA 7 software. The numbers at the nodes are percentages of bootstrap values obtained by repeating the analysis 1000 times to generate a majority consensus tree.

**Fig. 2:**
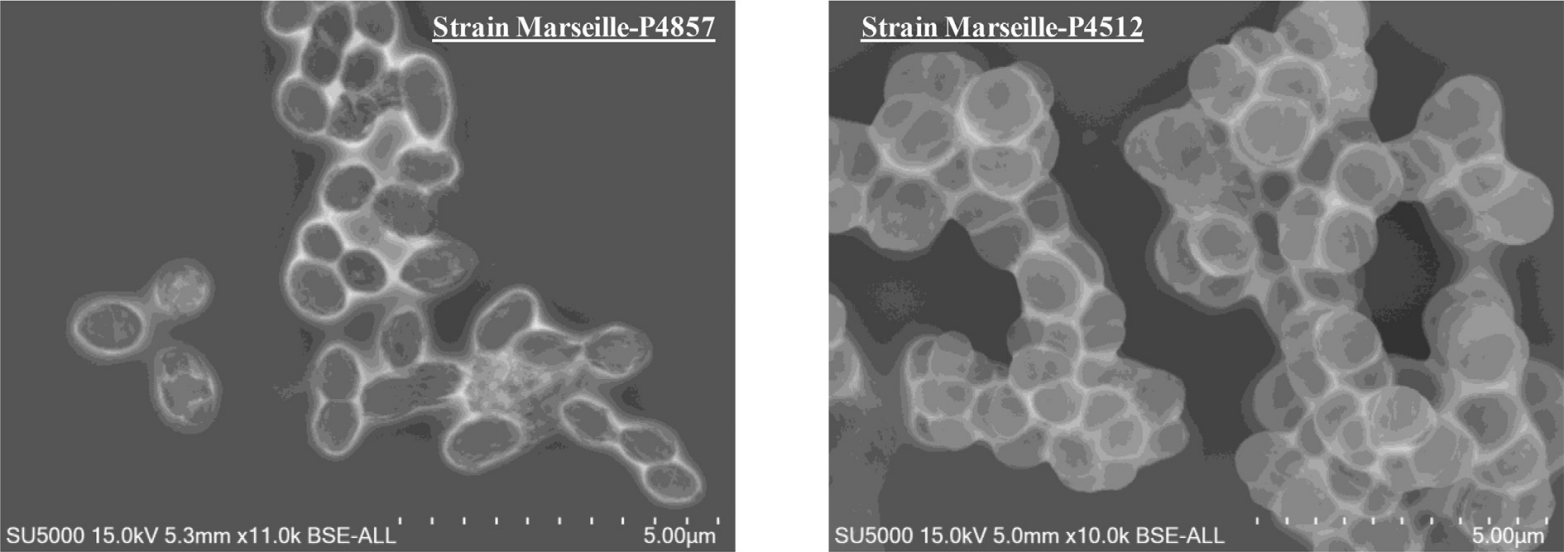
Scanning electron micrograph of *Megasphaera vaginalis* strain Marseille-P4857^T^ and *Anaerococcus vaginimassiliensis* strain Marseille-P4512^T^ using the scanning electron microscope TM4000 from Hitachi. Scale bar and acquisition settings are presented on the pictures.

Attempts to identify the strains cultivated on blood agar by mass spectrometry failed, indicating that these isolates were not known from the MALDI-TOF database. Therefore, their generated spectra were added to the local database. 16S rDNA-based similarity analysis of strain Marseille-P4857 and strain Marseille-P4512 against GenBank exhibited highest nucleotide sequence similarities of 95.12% with *Megasphaera micronuciformis* strain AIP 412.00 (accession number NR_025230.1) and 96.78% with *Anaerococcus tetradius* strain CCUG 46590 (accession number NR_041941.1), being respectively the two phylogenetically closest species. As these similarity values were below the 98.65% threshold recommended for the delimitation of new bacterial species [29,31], strain Marseille-P4857 and strain Marseille-P4512 were considered potentially new species within the phylum *Firmicutes*. The phylogenetic trees of *Megasphaera* spp. (Fig. 1a) and *Anaerococcus* spp. (Fig. 1b) show their positions concerning their respective closely related species with a validly published name. In addition, the shape of each bacterium (shown in Fig. 2) was obtained from the Hitachi TM4000 instrument.

### Biochemical properties of the strains

The two strains grow strictly under anaerobic conditions with an optimal temperature at 37°C. Strain Marseille-P4857 is a Gram-negative anaerobic coccus with a mean cell diameter of 0.70 μm. Colonies of strain Marseille-P4857 were white to yellow, shiny, opaque and convex with a diameter varying from 0.5 to 1 mm on blood agar after 3 days of incubation. It presents catalase-negative and oxidase-negative activities. Conversely, strain Marseille-P4512 is a Gram-positive anaerobic bacterium. Cells are coccoid with a mean diameter of 1.08 μm. They exhibit catalase-positive and oxidase-negative activities. Colonies of strain Marseille-P4512 are white with regular edges and a mean diameter of 2 mm.

Using the API ZYM strip, only acid phosphatase was positive for strain Marseille-P4857, while alkaline phosphatase, leucine arylamidase and acid phosphatase were also positive for strain Marseille-P4512. All remaining reactions were still negative with this API ZYM test. In addition, using the API 50 CH strip, *Megasphaera vaginalis* strain Marseille-P4857 was positive for glycerol, erythritol, arabinose, ribose, xylose, d-fructose, inositol, sorbitol, methyl αd-glucopyranoside, *N*-acetyl-glucosamine, amygdalin, arbutin, salicin, d-maltose, d-lactose, d-melibiose, sucrose, inulin, d-melezitose, d-raffinose, glycogen, xylitol, gentiobiose, d-lyxose, d-tagalose, fucose, potassium gluconate and potassium 5-ketogluconate. For *Anaerococcus vaginimassiliensis* strain Marseille-P4512, glycerol, xylose, galactose, fructose, glucose, methyl-αd-glucopyranoside, *N*-acetyl-glucosamine, amygdalin, arbutin, esculin ferric citrate, salicin, d-cellobiose, d-maltose, d-lactose, d-trehalose, xylitol, gentiobiose, potassium 5-ketogluconate were positive. A large phenotypic comparison of Marseille-P4857 and Marseille-P4512 with closely related species is displayed in Table 1, Table 2. The major fatty acids found for Marseille-P4857 were C_16:0_ (22%) and C_16:1n9_ (15%). Concerning Marseille-P4512, the major fatty acids were C_16:0_ (42%), C_18:1n9_ (25%) and C_18:2n6_ (19%). Minor amounts of saturated fatty acids were also detected for both.

**Table 1:** Different characteristics of *Megasphaera* species

Properties	1	2	3	4
Cell diameter (μm)	0.6–0.9	0.4–0.6	0.8	1.2–1.5
Oxygen requirement	—	—	—	—
Gram stain	—	—	—	—
Motility	—	—	—	—
Endospore formation	—	—	—	—
α-glucosidase	—	NA	+	NA
Catalase	—	—	—	—
Oxidase	—	NA	+	—
Glycerol	+	—	W	—
Erythritol	+	—	NA	—
d-arabinose	+	—	W	NA
l-arabinose	+	—	+	—
d-ribose	+	—	+	—
d-xylose	+	—	+	—
d-galactose	—	—	+	—
d-glucose	—	—	+	—
d-fructose	+	—	+	—
l-rhamnose	—	—	+	—
Dulcitol	—	—	NA	—
Inositol	+	—	NA	—
d-mannitol	—	—	+	—
d-sorbitol	+	—	+	NA
*N*-acetyl-glucosamine	+	—	+	—
Esculin ferric citrate	—	—	+	—
Salicin	+	—	+	—
d-cellobiose	—	—	+	—
d-maltose	+	—	+	—
d-lactose	+	—	+	—
d-melibiose	+	—	NA	—
d-trehalose	—	—	+	NA
d-melezitose	+	—	NA	—
d-raffinose	+	—	—	—
Glycogen	+	NA	NA	—
Source	human vaginal swab	human stool	human stool	spoiled beer

1, *Megasphaera vaginalis* sp. nov., strain Marseille-P4857; 2, *Megasphaera micronuciformis* strain AIP 412.00 [29]; 3, *Megasphaera massiliensis* strain NP3 [12]; 4, *Megasphaera paucivorans* strain DSM 16981 [15].

+, positive reaction; –, reaction; NA, not available data; w, weak reaction.

**Table 2:** Different characteristics of *Anaerococcus* species

Properties	1	2	3
Cell diameter (μm)	0.8–1.3	0.8–1.8	0.7–1.8
Oxygen requirement	—	—	—
Gram stain	+	+	+
Motility	—	—	—
Alkaline phosphatase	+	—	D
Leucine arylamidase	+	D	D
Acid phosphatase	+	NA	NA
α-galactosidase	—	—	+
β-galactosidase	—	—	D
β-glucuronidase	—	+	D
α-glucosidase	—	D	D
β-glucosidase	—	+	+
Catalase	+	D	D
Oxidase	—	NA	NA
Glycerol	+	NA	—
d-ribose	—	—	+
Xylose	+	—	—
d-glucose	+	+	D
d-fructose	+	+	D
d-maltose	+	D	D
d-lactose	+	—	—
Source	vaginal swab	vaginal discharge	vaginal discharge

1, *Anaerococcus vaginimassiliensis* strain Marseille-P4512; 2, *Anaerococcus tetradius* strain JCM 1964^T^ [16]; 3, *Anaerococcus prevotii* strain ATCC 9321^T^ [16].

+, positive reaction; –, reaction; NA, not available data; D, strain-dependent.

### Genomic analysis

The size of the genomes of strains Marseille-P4857 and Marseille-P4512 were 2 206 375 and 1 836 452 bp with 50.2 and 33.1 mol% G + C content, respectively. The genomic assembly was carried out into 17 contigs for Marseille-P4857 and into one scaffold for Marseille-P4512. Indeed, 2137 and 1826 were assigned as predicted genes for Marseille-P4857 and Marseille-P4512, respectively. In addition, 2032 and 1722 protein-coding genes and 56 and 61 RNA genes were found from the respective genomes of Marseille-P4857 and Marseille-P4512. The comparison of the genomes of *M. vaginalis* and *A. vaginimassiliensis* in terms of size and G + C content, as well as the number of genes compared with their phylogenetically closest species is presented in Table 3.

**Table 3:** Genome comparison of closely related species to *Megasphaera vaginalis* strain Marseille-P4857^T^ and *Anaerococcus vaginimassiliensis* strain Marseille-P4512^T^

Species	Size (Mb)	G + C mol%	Protein	rRNA	tRNA	Other RNA	Gene	Pseudogene
*Megasphaera vaginalis*	2.21	50.2	2032	7	49	4	2137	45
*Megasphaera cerevisiae*	3.24	44.8	2933	17	55	4	3163	154
*Megasphaera paucivorans*	2.91	40.2	2598	14	51	4	2780	113
*Megasphaera micronuciformis*	1.77	45.4	1665		48		1746	29
*Megasphaera elsdenii*	2.50	52.8	2211	21	65	4	2378	75
*Megasphaera stantonii*	2.65	52.6	2397	18	57	4	2509	33
*Megasphaera massiliensis*	2.74	50.2	2388	3	56	4	2562	111
*Megasphaera hexanoica*	2.88	49.0	2636	18	53	1	2750	42
*Anaerococcus vaginimassiliensis*	1.84	33.1	1722	13	48	3	1826	40
*Anaerococcus vaginalis*	1.89	29.0	1693	2	46	4	1793	48
*Anaerococcus mediterraneensis*	2.08	34.6	1936	9	44	4	2045	52
*Anaerococcus tetradius*	2.15	34.4	1895	5	45	4	2010	61
*Anaerococcus prevotii*	1.70	33.0	1563	3	44	3	1658	45
*Anaerococcus marasmi*	2.13	35.4	1953	14	49	4	2082	62
*Anaerococcus senegalensis*	1.80	28.6	1625	3	47	4	1756	77
*Anaerococcus provencensis*	2.27	33.7	2004	9	48	3	2146	82

Using dDDH analysis, values ranged from 17.7% between *M. massiliensis* and *Megasphaera paucivorans* to 27.0% between *M. micronuciformis* and *Megasphaera stantonii.* At the end of the dDDH analysis of *Anaerococcus* species used in this study, we obtained values ranging from 20.2% between *A. prevotii* ACS-065-V-Col13 and *Anaerococcus mediterraneensis* strain Marseille-P2765 to 33.6% between *A. vaginalis* ATCC 51170 and *A. mediterraneensis* strain Marseille-P2765. These values are lower than the 70% threshold used for the delineation of prokaryotic species, confirming that these three strains represent new species. The dDDH values obtained from genome analysis of the species studied here are shown in Table 4.

**Table 4:** Genomic comparison of *Megasphaera vaginalis* strain Marseille-P4857 and *Anaerococcus vaginimassiliensis* strain Marseille-P4512 between their closely related species using Genome-to-Genome Distance Calculator and formula 2 (dDDH estimates based on identities over HSP length)

% Similarity of *Megasphaera* species								
	MEL	MMI	MCE	MST	MHE	MPA	MMA	MVA
MEL	100							
MMI	26.0	100						
MCE	19.2	21.7	100					
MST	21.7	27.0	18.6	100				
MHE	23.7	24.8	19.5	21.0	100			
MPA	19.7	21.3	22.2	20.1	20.1	100		
MMA	24.3	20.0	18.2	20.5	20.2	17.7	100	
MVA	20.2	19.3	18.8	19.4	19.2	17.9	20.4	100
**% Similarity of *Anaerococcus* species**								

	**AVG**	**APR**	**AVA**	**ATE**	**APA**	**ASE**	**AME**	**APV**
AVG	100	21.4	27.1	21.9	25.9	21.7	23.4	22.6
APR		100	21.6	21.5	20.5	21.8	20.2	20.5
AVA			100	28.8	36.0	29.4	33.6	26.2
ATE				100	24.6	32.3	21.2	22.2
APA					100	25.7	25.5	21.7
ASE						100	24.3	21.6
AME							100	23.9
APV								100

Abbreviations: MEL, *Megasphaera elsdenii* 14-14 (NZ_CP009240.1); MMI, *Megasphaera micronuciformis* F0359 (NZ_AECS00000000.1); MCE, *Megasphaera cerevisiae* DSM 20462 (FUXD01000000); MST, *Megasphaera stantonii* DSM 106750 (NZ_CP029462.1); MHE, *Megasphaera hexanoica* MH (CP011940.1); MPA, *Megasphaera paucivorans* DSM 16981 (NZ_FNHQ00000000.1); MMA, *Megasphaera massiliensis* NP3 (CAVO000000000.1) and MVA, *Megasphaera vaginalis* Marseille-P4857 (NZ_OEQB00000000.1). AVG, *Anaerococcus vaginimassiliensis* Marseille-P4512 (UZAS00000000); APR, *Anaerococcus prevotii* ACS-065-V-Col13 (NC_013171.1); AVA, *Anaerococcus vaginalis* ATCC 51170 (NZ_CAGU00000000.1); ATE, *Anaerococcus tetradius* ATCC 35098 (ACGC00000000.1); APA, *Anaerococcus pacaensis* 9403502 (CAJJ000000000.2); ASE, *Anaerococcus senegalensis* JC48 (NZ_CAEK00000000.1); AME, *Anaerococcus mediterraneensis* Marseille-P2765 (NZ_LT635772.1); APV, *Anaerococcus provencensis* 9402080 (NZ_CAJU000000000.2).

**Fig. 3:**
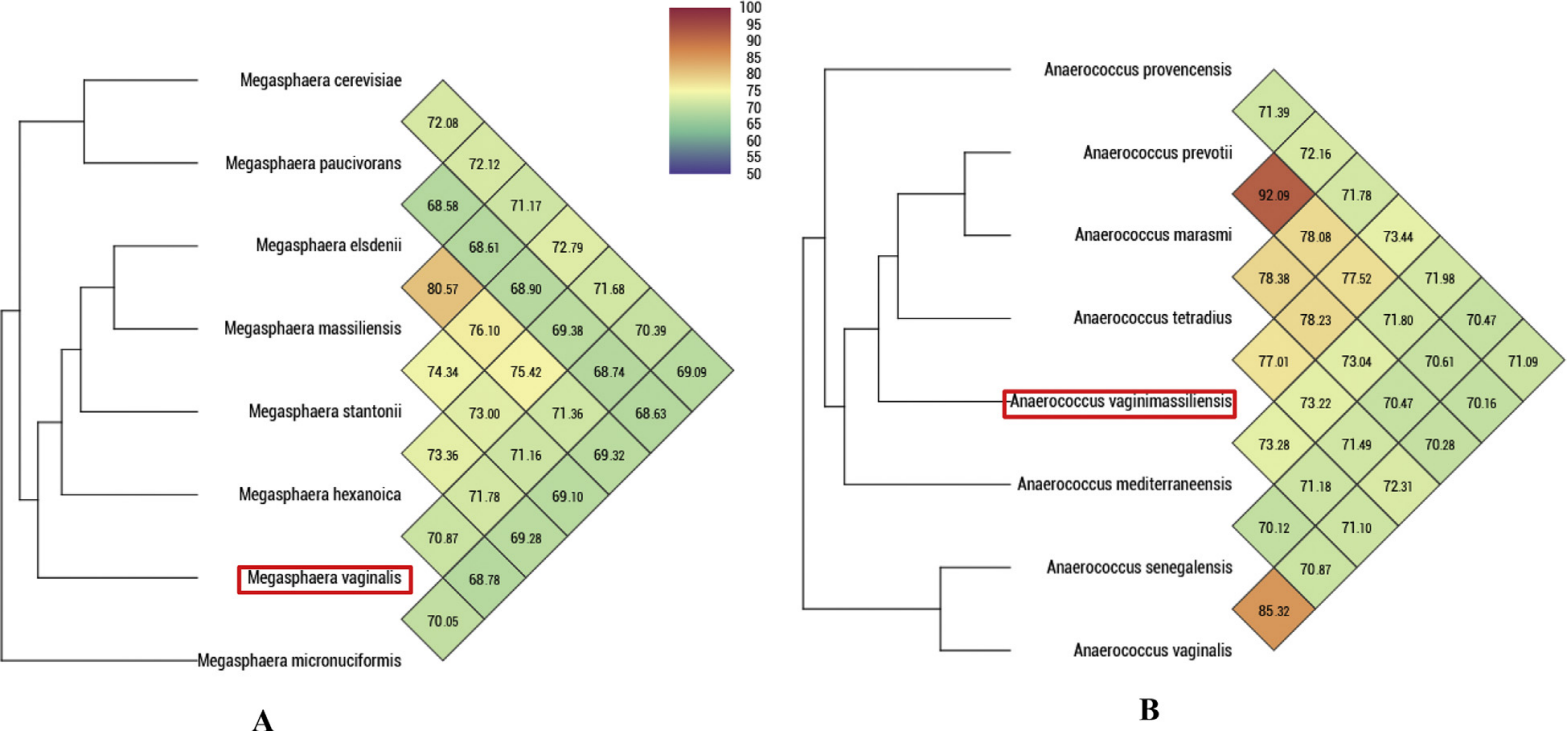
Heatmap generated with OrthoANI values calculated using the OAT software for *Megasphaera vaginalis* sp. nov., strain Marseille-P4857 (a) and *Anaerococcus vaginimassiliensis* strain Marseille-P4512 (b) with their respective closely related species with standing in nomenclature.

In addition, OrthoANI analysis among closely related species (Fig. 3) highlighted that *Megasphaera* species had a higher value of percentage of identity of 80.57% shared between *Megasphaera elsdenii* and *M. massiliensis*. The lowest value of similarity, 68.58%, was obtained between *M. elsdenii* and *M. paucivorans*. Hence, OrthoANI analysis for *Anaerococcus* species revealed that 71.78% was the highest value of similarity that the *M. vaginalis* Marseille-P4857 strain shared with *M. stantonii*. Analysis of *Anaerococcus* species revealed that OrthoANI values ranged from 92.09% of similarity with *A. prevotii* and *Anaerococcus marasmi* to 70.12% of similarity with *A. mediterraneensis* and *Anaerococcus senegalensis*. The highest percentage value obtained with strain Marseille-P4512 was 78.23% of similarity with *A. marasmi*.

## Conclusion

Considering the phenotypic, biochemical and genomic analysis carried out on these bacteria, strains Marseille-P4857 and Marseille-P4512 are proposed as new species. In addition, the genomic evidence used in this study, such as the sequence similarity of the 16S rRNA gene below the threshold value of 98.65% or OrthoANI values < 95% allowed us to formally declare that *Megasphaera vaginalis* sp. nov. and *Anaerococcus vaginimassiliensis* sp. nov., are new species within the phylum *Firmicutes*.

## Description of *Megasphaera vaginalis* sp. nov

*Megasphaera vaginalis* sp. nov. (va.gi.na'lis. L. n. fem. gen. *vaginalis* from the vagina which is a female genital organ; *vaginalis* referring to the vagina). This bacterium is Gram-negative, anaerobic and shell-shaped. Cells are 0.62–0.91 μm in diameter. Catalase and oxidase activities are negative. Acid phosphatase activity is present. Colonies are white, shiny and convex with a mean diameter of 0.5 mm on blood agar. The following tests were positive: glycerol, erythritol, arabinose, ribose, d-xylose, d-fructose, inositol, d-sorbitol, methyl αd-glucopyranoside, *N*-acetyl-glucosamine, amygdalin, arbutin, salicin, sucrose, inulin, d-maltose, d-lactose, d-melibiose, d-melezitose, d-raffinose, glycogen, xylitol, gentiobiose, d-lyxose, d-tagalose, d-fucose, l-fucose, potassium gluconate and potassium 5-ketogluconate. C_16:0_ (22.0%), C_16:1n9_ (14.8%), C_12:0_ (9.0%) and C_14:0 3-OH_ (7.3%) were the major fatty acids found with *Megasphaera vaginalis* sp. nov. The genome of strain Marseille-P4857 was 2.20 Mbp with 50.2 mol% of G + C content. The 16S rRNA and draft genome sequences are deposited in the Genbank database under Accession numbers LT960586 and OEQB00000000, respectively. The type strain of *Megasphaera vaginalis* sp. nov., strain Marseille-P4857 was isolated from the vagina of a woman with bacterial vaginosis.

## Description of *Anaerococcus vaginimassiliensis* sp. nov

*Anaerococcus vaginimassiliensis* sp. nov. (va.gi.ni.mas.si.li.en'sis N.L. fem. adj. *vaginimassiliensis*: *vagini* refers to vagina and *massiliensis* to Massilia, the Latin name of Marseille where the type strain was isolated). Gram-staining is positive. It is a coccus-shaped bacterium with a diameter ranged from 0.8 to 1.2 μm. *Anaerococcus vaginimassiliensis* sp. nov., is a strict anaerobic bacterium that grows preferentially at 37°C. It has catalase activity, but not oxidase. Colonies are white with regular boundaries and have a mean diameter of 2 mm. The *A. vaginimassiliensis* is able to ferment glycerol, xylose, d-galactose, d-glucose, d-fructose, methyl αd-glucopyranoside, *N*-acetyl-glucosamine, amygdalin, arbutin, esculin ferric citrate, salicin, trehalose, cellobiose, maltose, lactose, xylitol, gentiobiose and potassium 5-ketogluconate. Alkaline phosphatase, leucine arylamidase and acid phosphatase are positive. The major fatty acids were C_16:0_ (42%), C_18:1n9_ (25%) and C_18:2n6_ (19%). The genome size of *A. vaginimassiliensis* strain Marseille-P4512 is 1.83 Mbp with 33.1 mol% G + C content. The 16S rRNA and draft genome sequences of strain Marseille-P4512, are available in GenBank database under accession numbers LT934505 and UZAS00000000, respectively. The type strain is Marseille-P4512^T^, which was isolated from the vagina of a woman with bacterial vaginosis.

## Funding

Effectively, our study is supported by the Institut Hospitalo-Universitaire (IHU) Méditerranée Infection, the 10.13039/501100001665National Research Agency under the program Investissements d’avenir, reference ANR-10-IAHU-03, the Région Provence Alpes Côte d’Azur and European funding FEDER PRIMI.

## Conflict of interest

Authors declare that there are not conflict of interest.

## References

[1] Mitra A., MacIntyre D.A., Lee Y.S., Smith A., Marchesi J.R., Lehne B. (2015). Cervical intraepithelial neoplasia disease progression is associated with increased vaginal microbiome diversity. Sci Rep.

[2] Mitra A., MacIntyre D.A., Marchesi J.R., Lee Y.S., Bennett P.R., Kyrgiou M. (2016). The vaginal microbiota, human papillomavirus infection and cervical intraepithelial neoplasia: what do we know and where are we going next?. Microbiome.

[3] Ghartey J.P., Smith B.C., Chen Z., Buckley N., Lo Y., Ratner A.J. (2014). Lactobacillus crispatus dominant vaginal microbiome is associated with inhibitory activity of female genital tract secretions against Escherichia coli. PLoS One.

[4] Aldunate M., Srbinovski D., Hearps A.C., Catherine F.L., Paul A.R., Raffi G. (2015). Antimicrobial and immune modulatory effects of lactic acid and short chain fatty acids produced by vaginal microbiota associated with eubiosis and bacterial vaginosis. Front Physiol.

[5] Lagier J.C., Hugon P., Khelaifia S., Fournier P.E., La Scola B., Raoult D. (2015). The rebirth of culture in microbiology through the example of culturomics to study human gut microbiota. Clin Microbiol Rev.

[6] Sankar S.A., Lagier J.C., Pontarotti P., Raoult D., Fournier P.E. (2015). The human gut microbiome, a taxonomic conundrum. Syst Appl Microbiol.

[7] Diop K., Diop A., Khelaifia S., Robert C., Di Pinto F., Delerce J. (2018). Characterization of a novel Gram-stain-positive anaerobic coccus isolated from the female genital tract: Genome sequence and description of Murdochiella vaginalis sp. nov. Microbiologyopen.

[8] Diop K., Diop A., Levasseur A., Mediannikov O., Robert C., Armstrong N. (2018). Microbial Culturomics Broadens Human Vaginal Flora Diversity: Genome Sequence and Description of Prevotella lascolaii sp. nov., isolated from a Patient with Bacterial Vaginosis. OMICS.

[9] Diop K., Diop A., Bretelle F., Cadoret F., Michelle C., Richez M. (2017). Olegusella massiliensis gen. nov., sp. nov., strain KHD7T, a new bacterial genus isolated from the female genital tract of a patient with bacterial vaginosis. Anaerobe.

[10] Parte A.C. (2018). LPSN - List of Prokaryotic names with Standing in Nomenclature (bacterio.net), 20 years on. Int J Syst Evol Microbiol.

[11] Rogosa M. (1971). Transfer of Peptostreptococcus elsdenii Gutierrez et al. to a New Genus, Megasphaera [M. elsdenii (Gutierrez et al.) comb. nov.]. Int J Syst Evol Microbiol.

[12] Padmanabhan R., Lagier J.C., Dangui N.P., Michelle C., Couderc C., Raoult D. (2013). Non-contiguous finished genome sequence and description of Megasphaera massiliensis sp. nov. Stand Genomic Sci.

[13] Lanjekar V.B., Marathe N.P., Ramana V.V., Shouche Y.S., Ranade D.R. (2014). Megasphaera indica sp. nov., an obligate anaerobic bacteria isolated from human faeces. Int J Syst Evol Microbiol.

[14] Maki J.J., Looft T. (2018). Megasphaera stantonii sp. nov., a butyrate-producing bacterium isolated from the cecum of a healthy chicken. Int J Syst Evol Microbiol.

[15] Juvonen R., Suihko M.L. (2006). Megasphaera paucivorans sp. nov., Megasphaera sueciensis sp. nov. and Pectinatus haikarae sp. nov., isolated from brewery samples, and emended description of the genus Pectinatus. Int J Syst Evol Microbiol.

[16] Ezaki T., Kawamura Y., Li N., Li Z.Y., Zhao L., Shu S. (2001). Proposal of the genera Anaerococcus gen. nov., Peptoniphilus gen. nov. and Gallicola gen. nov. for members of the genus Peptostreptococcus. Int J Syst Evol Microbiol.

[17] Marchandin H., Jumas-Bilak E., Gay B., Teyssier C., Jean-Pierre H., Siméon de Buochberg M. (2003). Phylogenetic analysis of some Sporomusa sub-branch members isolated from human clinical specimens: description of Megasphaera micronuciformis sp. nov. Int J Syst Evol Microbiol.

[18] Pagnier I., Croce O., Robert C., Raoult D., La Scola B. (2014). Non-contiguous finished genome sequence and description of Anaerococcus provenciensis sp. nov. Stand Genomic Sci.

[19] Lo C.I., Fall B., Sambe-Ba B., Diawara S., Gueye M.W., Mediannikov O. (2015). MALDI-TOF Mass Spectrometry: A Powerful Tool for Clinical Microbiology at Hôpital Principal de Dakar, Senegal (West Africa). PLoS One.

[20] Morel A.-S., Dubourg G., Prudent E., Edouard S., Gouriet F., Casalta J.P. (2015). Complementarity between targeted real-time specific PCR and conventional broad-range 16S rDNA PCR in the syndrome-driven diagnosis of infectious diseases. Eur J Clin Microbiol Infect Dis.

[21] Wormser G.P., Stratton C., Murray P.R., Baron E.J., Jorgensen J.H., Landry M.L., Pfaller M.A. (2007). Manual of clinical microbiology.

[22] Belkacemi S., Bou K.J., Ominami Y., Hisada A., Fontanini A., Caputo A. (2019). Passive filtration, rapid scanning electron microscopy, and matrix-assisted laser desorption ionization-time of flight mass spectrometry for *Treponema* culture and identification from the oral cavity. J Clin Microbiol.

[23] Zerbino D.R., Birney E. (2008). Velvet: algorithms for de novo short read assembly using de Bruijn graphs. Genome Res.

[24] Bankevich A., Nurk S., Antipov D., Gurevich A.A., Dvorkin M., Kulikov A.S. (2012). SPAdes: a new genome assembly algorithm and its applications to single-cell sequencing. J Comput Biol.

[25] Luo R., Liu B., Xie Y., Li Z., Huang W., Yuan J. (2012). SOAPdenovo2: an empirically improved memory-efficient short-read de novo assembler. Gigascience.

[26] Bolger A.M., Lohse M., Usadel B. (2014). Trimmomatic: a flexible trimmer for Illumina sequence data. Bioinformatics.

[27] Xu G.C., Xu T.J., Zhu R., Zhang Y., Li S.Q., Wang H.W. (2019). LR_Gapcloser: a tiling path-based gap closer that uses long reads to complete genome assembly. Gigascience.

[28] Lo C.I., Sankar S.A., Fall B., Sambe-Ba B., Diawara S., Gueye M.W. (2016). High-quality draft genome sequence and description of *Haemophilus massiliensis* sp. nov. Stand Genomic Sci.

[29] Meier-Kolthoff J.P., Auch A.F., Klenk H.P., Göker M. (2013). Genome sequence-based species delimitation with confidence intervals and improved distance functions. BMC Bioinform.

[30] Lee I., Ouk Kim Y., Park S.C., Chun J. (2016). OrthoANI: an improved algorithm and software for calculating average nucleotide identity. Int J Syst Evol Microbiol.

[31] Kim M., Oh H.S., Park S.C., Chun J. (2014). Towards a taxonomic coherence between average nucleotide identity and 16S rRNA gene sequence similarity for species demarcation of prokaryotes [published correction appears in Int J Syst Evol Microbiol. Int J Syst Evol Microbiol.

